# Putative Loci Causing Early Embryonic Mortality in Duroc Swine

**DOI:** 10.3389/fgene.2018.00655

**Published:** 2018-12-17

**Authors:** Chunyan Zhang, Michael D. MacNeil, Robert A. Kemp, Michael K. Dyck, Graham S. Plastow

**Affiliations:** ^1^Department of Agricultural, Food & Nutritional Sciences, University of Alberta, Edmonton, AB, Canada; ^2^Delta G, Miles City, MT, United States; ^3^Department of Animal, Wildlife and Grassland Sciences, University of the Free State, Bloemfontein, South Africa; ^4^Genesus Inc., Oakville, MB, Canada

**Keywords:** Duroc pigs, lethal recessive loci, fertility, haplotype blocks, 650K SNP chip

## Abstract

Lethal recessive alleles that act prenatally may be detected from the absence of homozygous individuals in a population. However, these alleles may be maintained at relatively low frequencies in populations as heterozygotes. In pigs, they may reduce litter size. This study aimed to detect putative lethal variants in the Duroc breed. Phenotypes for the numbers of piglets born (TNB), born live (BA), alive at 24 h (L24), stillborn (SB), and born as mummified fetuses (MM) were available from 5340 recorded litters which resulted from mating of 192 genotyped boars with sows of unknown genotype (dataset 1). An additional 50 litters were produced from parents that were both genotyped (dataset 2). Imputed genotypes of 650K SNPs for 1359 Duroc boars were used in this study. One significant SNP (Bonferroni corrected *P* = 5.5E-06) was located on SSC14 with 45.3 homozygous individuals expected but none observed. This SNP was significant for mummified fetuses. One hundred fifty two haplotypes were also found to potentially harbor recessive lethal mutations. Twenty-one haplotypes had a significant harmful effect on at least one trait. Two regions, located on SSC8 (144.9–145.5 Mb) and SSC9 (19–19.4 Mb) had significant effects on fertility traits in both datasets. Additionally, regions on SSC1 (82.0–82.8 Mb), SSC3 (73.3–73.7 and 87.1–87.5 Mb) and SSC12 (35.8–36.2 and 50.0–50.5 Mb) had significant deleterious effects on TNB or BA or L24 in dataset 1. Finally, a region on SSC17 (28.7–29.3 Mb) had significant effects on TNB, BA and L24 in dataset 2. A few candidate genes identified within these regions were described as being involved in spermatogenesis and male fertility (*TEX14, SEP4*, and *HSF5*), or displayed recessive lethality (*CYP26B1*, *SCD5*, and *PCF11*) in other species. The putative loci detected in this study provide valuable information to potentially increase Duroc litter size by avoiding carrier-by-carrier matings in breeding programs. Further study of the identified candidate genes responsible for such lethal effects may lead to new insights into functions regulating pig fertility.

## Introduction

Comparatively small litters are characteristic of the Duroc breed (Johnson, 1981; Sonderman and Luebbe, 2008). Yet, use of Duroc as a terminal sire is a mainstay of swine production due to favorable direct effects on growth and carcass traits (Johnson, 1981; Zhang et al., 2016). Early embryonic mortality, affecting litter size, is recognized as a significant issue in swine (Bolet, 1986; Christianson, 1992). Geisert and Schmitt (2002) noted a major limitation for increasing litter size in swine is embryonic loss that occurs during the second to third week of gestation. Many factors may contribute to this loss and most work has focused on increasing litter size in the maternal lines based on Landrace and Large White breeds where high ovulation rates exist and litter size is relatively high and responds to selection (Sorensen et al., 2000; Noguera et al., 2002). However, little is known about ovulation rate in Duroc sire lines and there has been little selection for increased litter size in this breed as the focus of genetic improvement programs has been on performance and carcass quality (Lonergan et al., 2001). One possible cause of low litter size in the Duroc may be the existence of recessive lethal mutations which result in embryonic loss. With the generation of large amounts of genomic data this hypothesis can now be tested where loci harboring lethal recessive alleles may be discovered from the absence of live animals that are homozygous. Precedent for this proposed mechanism is found in recent studies based on observations of missing homozygous haplotypes in cattle (VanRaden et al., 2011; Hoff et al., 2017; Howard et al., 2017). Only genotypes from apparently normal individuals and not from affected embryos are required. Thus, under the assumptions that neither ovulation rate (Young et al., 1976) nor uterine capacity prior to fetal implantation are limiting in Duroc, we hypothesized that early embryonic mortality due to homozygosity of recessive lethal alleles may contribute to the production of relatively small litters by Duroc females. Due to the low reproductive rate of Duroc as a purebred there is: (1) an immediate cost due to a reduction in number of pigs produced per litter; and (2) a recurrent cost due to a decreased rate of genetic improvement resulting from reduced selection intensity. Therefore, the primary objective of this research was to scan the Duroc genome for segregating individual single nucleotide polymorphisms (SNPs) and haplotypes for which one homozygous genotype is either absent or in unexpectedly low frequency. A secondary objective was to test the association of any candidate loci with phenotypes (total number of born, number of born alive, number alive at 24 h, stillborn piglets, and number of mummified fetuses) that are related to fertility of swine. The third objective was to identify some candidate genes that might be involved in functional pathways of sow fertility.

## Materials and Methods

DNA from 1359 Duroc boars born between 2012 and 2013 was genotyped using the Geneseek-Neogen GPPHD 80K SNP chip^1^. The pedigree of these boars was used to identify 171 of their sires and grandsires. DNA from these ancestors was genotyped using the Affymetrix Axiom^®^ 650K SNP Array. DNA extraction and genotyping were conducted at the Delta Genomics laboratory (Edmonton, AB, Canada). The genotypes and details of genotyping and imputation were described by Zhang et al. (2018). Briefly, average imputation accuracy (percentage of correctly imputed SNPs) from 80 to 650K was 92.1% and only those SNPs with accuracy of imputation ≥ 95% were used in this study. Additional quality control standards included: animal call rate ≥ 95%, SNP call rate ≥ 95%, SNPs mapped to the autosomal chromosomes (*Sscrofa10.2*), minor allele frequency (MAF) ≥ 5%, and Chi square of Hardy–Weinberg equilibrium test less than 600. After editing, a total of 416,392 SNPs were used for this study.

### Detection of an Absent Homozygous Genotype

For each SNP, the expected number of animals with a homozygous genotype was calculated assuming random mating and determined as the number of genotyped animals multiplied by the square of the MAF. Those SNPs with greater than five individuals expected to be homozygous for the minor allele, but where none were observed in the data, were considered to be necessary for life. Further, the difference between the observed and expected number of homozygotes was tested using a Chi-squared test (df = 1). The Bonferroni-corrected significance threshold was set as *P < 0.01*.

Genotypes were phased on each chromosome using SHAPEIT2 (Delaneau et al., 2012, 2014) with an average window size of 2 Mb and effective population size of 75 as recently reported for Canadian Duroc by Grossi et al. (2017). As there is no complete genetic map for pigs, a consistent recombination rate was given for each chromosome according to the average value from Tortereau et al. (2012). The phased genotypes were used to construct haplotype blocks and detect blocks containing potential recessive lethal alleles using GHap (Utsunomiya et al., 2016). The optimal window size was defined as 400 Kb with a sliding block of 100 Kb, following Veroneze et al. (2013), in which it was reported that the average extent of linkage disequilibrium (LD) in pig was about 400 Kb.

The expected number of animals with a homozygous haplotype within each window was calculated assuming random mating as no direct selection for litter size occurred in this population and determined as the number of genotyped animals divided by 4 and multiplied by the square of the carrier frequency of that particular haplotype (VanRaden et al., 2011). The blocks with 3 SNPs or less, or haplotype alleles with MAF < 0.01 were excluded. The difference between the observed and expected number of homozygotes was again tested by Chi-squared of Hardy–Weinberg equilibrium distribution. The *P*-value from the Chi-square test was adjusted for whole genome wide significance by Bonferroni correction. A haplotype allele was defined as a putative recessive lethal if the observed number of homozygotes was zero and the expected number of homozygotes was greater than 6 as suggested by previous studies (Sahana et al., 2013; Haggman and Uimari, 2017), and a Bonferroni corrected *P*-value < 0.01.

### Association Analysis

Between 2006 and 2016, 192 genotyped boars sired 5340 recorded litters from the sows with unknown genotypes (dataset 1). Five traits: numbers born (TNB), born alive (BA), alive at 24 h (L24), stillborn piglets (SB), and mummified fetuses (MM) were used in association analyses to evaluate the effect of the detected haplotypes on fertility. Although they were count data, the distributions of TNB, BA and L24 were approximately normal and they were analyzed in linear animal models in ASREML (Gilmour et al., 2008). The distributions of SB and MM were Poisson and a Poisson regression animal model in the MCMCglmm R package (Hadfield, 2010) was used for their association analyses. The model was described as below.

y = Xb + Mi + Za + Wv + e

In this model, *y* is the observed phenotype, ***X*** is an incidence matrix associated with fixed effects of parity (1, 2, 3, 4, 5, >6) (not included for MM) and farrowing year-season (37 levels), ***b*** is the vector of those fixed effects, ***M*** is the design matrix of haplotype alleles (0 = non-carrier, 1 = carrier), *i* is the vector of corresponding haplotype allele effect on observation (linear regression) or log of observation (Poisson regression), ***Z*** is an incidence matrix indicating random additive genetic effects (*a*) due to boars with mean 0 and (co)variance structure of *A*$\sigma_{a}^{2}$ where ***A*** is the pedigree-based additive relationship matrix and $\sigma_{a}^{2}$ is the additive genetic variance, ***W*** is an incidence matrix indicating random effects (*v*) due to sows with (co)variance structure of *I*$\sigma_{e}^{2}$ where *I* is identical matrix and $\sigma_{e}^{2}$ is sow random variance, and ***e*** is a vector of random residual errors.

Additionally, there were 50 litters with known mating types which were generated from 27 genotyped boars mated with 22 genotyped sows (dataset 2). This information was suitable to estimate the effect of the lethal haplotype combination on TNB, BA an L24, but not for SB or MM due to small numbers of observations. Mating type for each detected lethal haplotype allele, including carrier mated with carrier (11), non-carrier mated with non-carrier (00) and carrier mated with non-carrier (10 or 01), was fitted as a fixed effect in the above linear model (***M_i_***) to test their effect and significance. Due to the small sample size, the population mean was the only fixed effect that was included in the model. The significant threshold was set as *P* < 0.10.

### Candidate Gene Identification

Genes located within or flanking a detected significant haplotype block by less than 400 Kb were identified using pig genome assembly of *Sscrofa 10.2*.^2^ Initial identification of candidate genes was based on the annotated functions and related information that has been summarized in the GENECARDS^3^ database. Only the genes which were related to fertility functional pathways were reported and discussed in this paper.

## Results and Discussion

### Single SNP and Haplotype Detection

Only one SNP was found to have a sufficient expected number of homozygous recessive individuals with none being observed to attain statistical significance (Bonferroni corrected *P* = 5.5E-06). It had a MAF of 0.17 and thus it was expected that 45.3 homozygous recessive individuals would have been found. This SNP showed an effect on MM that litters sired by the boars carrying such allele had an average of 0.15 more log of mummified fetuses than the litters sired by non-carrier boars (*P* = 0.09). This SNP was on SSC14 at 59.5 Mb. Howard et al. (2017) observed a haplotype at 60.26 – 60.62 Mb on SSC 14 that was significantly associated with TNB in Large White.

A total of 152 haplotypes from 129 blocks were detected as potentially harboring recessive lethal mutations with Bonferroni-corrected *P* < 0.01. The SNP on SSC14 identified above was contained within one of these haplotypes. Twenty-one of these haplotypes also had significant effects on at least one fertility trait (see below) and were presented in Table 1. There were 131 additional haplotypes that were deficient of a homozygous genotype for the minor allele (Supplementary Table S1). However, none of these latter haplotypes had a detectable effect on any of the fertility traits. These haplotypes are located on all autosomes except for SSC13 and SSC18. Each haplotype contained, on average, 32.6 (5 – 85) SNPs. The carrier frequency for these haplotypes ranged from 0.10 to 0.26 and the expected number of homozygous individuals varied from 17 to 102.

**Table 1:** Location and minor allele frequency for haplotypes which were expected to have two homozygous genotypes, but with only one found in the data and which had a significant effect on at least one of the fertility phenotypes.

SSC	Locus (Mb)	# SNPs^1^	Haplotype^2^	Frequency	E(HOM)^3^	P^4^
1	82.0–82.4	23	1	0.11	17	6.4E-03
1	82.4–82.8	11	2	0.18	48	2.4E-16
3	73.3–73.7	36	3	0.11	18	3.3E-03
3	87.1–87.5	16	4	0.12	20	2.9E-04
4	27.3–27.7	35	5	0.11	20	5.1E-04
4	27.3–27.7	35	6	0.11	18	3.7E-03
4	28.4–28.8	39	7	0.11	19	1.1E-03
5	20.1–20.5	13	8	0.14	28	1.0E-07
7	84.2–84.6	25	9	0.13	25	1.7E-06
8	144.9–145.3	40	10	0.11	18	3.7E-03
8	145.1–145.5	52	11	0.11	18	2.7E-03
9	15.3–15.7	26	12	0.15	33	1.3E-09
9	19.0–19.4	22	13	0.13	25	2.5E-06
9	126.9–127.3	28	14	0.17	45	3.9E-15
10	61.8–62.2	32	15	0.16	38	5.5E-12
12	35.8–36.2	72	16	0.12	21	1.1E-04
12	50.0–50.4	39	17	0.14	31	4.8E-09
12	50.1–50.5	53	18	0.13	24	4.7E-06
16	57.2–57.6	22	19	0.14	31	4.8E-09
17	28.7–29.1	8	20	0.12	22	6.2E-05
17	28.9–29.3	13	21	0.19	55	3.9E-19


*^*1*^Number of SNPs in the haplotype blocks; ^*2*^Haplotype index; ^*3*^number of individuals that were expected to be homozygous for the minor allele; ^*4*^Bonferroni corrected*

A few recent studies report potential harmful recessive haplotypes in pigs. Haggman and Uimari (2017) detected 26 putative lethal haplotypes in Finnish Yorkshire AI boars, with the most important one was located on SSC8 at 107.0 – 113.3 Mb which was significantly associated with number of stillborn piglets in first and later parities. In this study we detected three regions on SSC8 although they were all distant to the region detected in the Finnish Yorkshire AI boars. These regions included 144.9–145.5 Mb (Haplotypes 10–11), 28.3–28.7 Mb (Haplotype 80) and 49.5–49.9 Mb (Haplotype 81). The first region (Haplotypes 10–11) showed a significant effect on TNB and L24 which will be discussed below. Two other regions detected in this study, 46.5 – 47.3 Mb on SSC4 (Haplotypes 52–54) and 110.1 – 110.6 Mb on SSC15 (Haplotypes 120–122), were close to regions detected by Haggman and Uimari (2017) at SSC4: 48.33 – 50.61 Mb and SSC15: 111.32 – 113.19 Mb. A different study by Howard et al. (2017) found six regions containing putative lethal recessive haplotypes that significantly affected TNB in Large White pigs. Three of them were the same or very close to the regions we detected here, including SSC1: 137 Mb, SSC10: 29 Mb, and SSC14: 60 Mb. In our study, the latter region on SSC14 contained the most significant SNP (14_59487297) detected by single SNP analyses. A third study by Derks et al. (2017) detected 145 haplotypes and 35 of them showed a negative effect on at least one of the analyzed reproductive traits. The region on SSC14 at 146 – 150 Mb was also detected in our study (Supplementary Table S1) although it was not found to have an impact on fertility. Other important regions detected in our study include SSC4:74.7 – 75.1 Mb (Haplotype 56), SSC6:111 Mb (Haplotypes 66–68), SSC9:127 Mb (Haplotypes 84–85) and SSC12:36Mb (Haplotype 16, Haplotypes 97–98), which were reported as harboring QTL for ovulation rate, number of viable embryos and embryo survival (Bidanel et al., 2008).

### Associations With Fertility Traits

A total of 192 boars were assumed to be randomly mated with 3460 sows with unknown genotypes to generate 5340 litters (dataset 1). Nineteen haplotypes were significant for at least one fertility trait (Tables 2, 3). Haplotypes on SSC 3 at 87.1–87.5 Mb, SSC 8 at 145.1–145.5 Mb, and SSC 9 at 19.0–19.4 Mb affected both TNB (*P* < 0.05) and either BA or L24 (*P* < 0.10). In each case, litters sired by carrier boars had approximately 0.3 fewer piglets than those sired by boars that were not carriers of the recessive haplotype. The two overlapping haplotypes on SSC 8 spanning the interval from 144.9–145.5 both affected TNB. Haggman and Uimari (2017) detected one recessive haplotype at 107.0 – 113.3 Mb on SSC8 in Finnish Yorkshire which showed a significant effect on number of stillborn piglets in both first and later parities. Three distinct haplotypes on SSC9 affected at least one trait.

**Table 2:** Effect (*P <* 0.10) of sire haplotype (non-carrier = 0, carrier = 1) on numbers of piglets born (TNB), born live (BA) and alive at 24 h postpartum (L24) as expressed in litters of purebred Duroc pigs.

Haplotype	SSC	Locus (Mb)	Trait^1^	Sire haplotype	
				0	1
2	1	82.4–82.8	TNB	10.51 ± 0.17	10.32 ± 0.18
3	3	73.3–73.7	L24	9.04 ± 0.17	8.80 ± 0.20
4	3	87.1–87.5	TNB	10.49 ± 0.17	10.18 ± 0.20
			BA	9.32 ± 0.19	9.08 ± 0.22
10	8	144.9–145.3	TNB	10.51 ± 0.18	10.28 ± 0.19
11	8	145.1–145.5	TNB	10.53 ± 0.18	10.25 ± 0.19
			L24	9.11 ± 0.17	8.82 ± 0.19
12	9	15.3–15.7	L24	9.08 ± 0.17	8.88 ± 0.18
13	9	19–19.4	TNB	10.53 ± 0.18	10.24 ± 0.19
			BA	9.36 ± 0.19	9.11 ± 0.21
16	12	35.8–36.2	L24	9.05 ± 0.17	8.82 ± 0.20
18	12	50.1–50.5	TNB	10.53 ± 0.18	10.25 ± 0.19


**Table 3:** Estimated difference between sire haplotypes (non-carrier – carrier) on numbers of stillborn piglets (SB) and mummified fetuses (MM) as expressed in litters of purebred Duroc pigs.

Haplotype	SSC	Locus (Mb)	Trait	Difference (log of count)	*P*
1	1	82.0–82.4	SB	-0.15 ± 0.07	0.046
5	4	27.3–27.7	MM	-0.20 ± 0.09	0.027
6	4	27.3–27.7	MM	-0.20 ± 0.09	0.024
7	4	28.4–28.8	MM	-0.18 ± 0.10	0.057
8	5	20.1–20.5	MM	-0.19 ± 0.10	0.051
9	7	84.2–84.6	MM	-0.15 ± 0.09	0.099
12	9	15.3–15.7	MM	-0.17 ± 0.10	0.079
14	9	126.9–127.3	MM	-0.16 ± 0.08	0.065
15	10	144.9–145.3	MM	-0.31 ± 0.10	0.002
17	12	126.9–127.3	MM	-0.17 ± 0.10	0.100
19	16	57.2–57.6	SB	-0.16 ± 0.05	0.001


Several additional loci were detected as having effects on one of the fertility traits. An additional haplotype on SSC 8 at 73.3–73.7 Mb was associated with L24 with carrier boars siring litters with 0.24 fewer piglets that were alive at 24 h postpartum. Three haplotypes on SSC 4 at approximately 28 Mb were each associated with the number of MM. Two distinct haplotypes, in addition to the locus described above, at 126.9–127.3 and 15.3–15.7 Mb affected numbers of MM, with the latter haplotype also affecting the number of piglets L24. Two adjacent haplotypes on SSC 1 at approximately 82 Mb were found to affect TNB or SB. Haplotypes on SSC12 at 35.8–36.2 and 50.1–50.5 Mb effected L24 and TNB, respectively. A QTL at approximately 31 Mb was previously reported for number of embryos (Bidanel et al., 2008). Further downstream at 126.9–127.3 Mb there was another haplotype that affected the number of MM.

Previously, two regions on SSC 1 at approximately 137 and 187 Mb were found to have significant effect on TNB in Large White (Howard et al., 2017). Eight haplotypes whose homozygotes for their recessive alleles were missing in the present study support the result of Howard et al. (2017). However, effects on the fertility phenotypes were not detected here (Supplementary Table S1).

Fifty litters were generated from matings between boars and sows with known genotypes (dataset 2). In this smaller dataset, four haplotypes affected (*P* < 0.10) at least one fertility trait (Table 4). The haplotypes on SSC 8 at 145.1–145.5 Mb and on SSC 9 at 19–19.4 Mb that were identified above again affected the numbers TNB and L24, respectively. In addition, the overlapping haplotypes located on SSC 17 at 28.7–29.1 and 28.9–29.3 Mb were significant for the three litter size traits. For the latter haplotype, litters from non-carrier mated with non-carrier had 1.96, 2.13, and 2.32 more piglets born, born live and alive at 24 h post-partum, respectively, than litters from the mating of carriers.

**Table 4:** Effects of mating type (non-carrier x non-carrier = 00, non-carrier × carrier = 01, carrier × carrier = 11) on TNB, BA and L24 as expressed in litters of purebred Duroc pigs.

Haplotype	SSC	Locus (Mb)	No.^1^		Mating type		
				Trait^2^	00	01	11
11	8	145.1-145.5	20/19/11	TNB	11.71 ± 0.54	9.84 ± 0.54	10.14 ± 0.72
13	9	19-19.4	15/24/11	L24	10.59 ± 0.70	9.83 ± 0.57	8.57 ± 0.74
20	17	28.7-29.1	27/20/3	BA	10.12 ± 0.43	9.86 ± 0.49	7.19 ± 1.26
				L24	10.09 ± 0.42	9.65 ± 0.48	7.18 ± 1.23
21	17	28.9-29.3	17/20/13	TNB	11.59 ± 0.56	10.51 ± 0.52	9.63 ± 0.65
				BA	10.87 ± 0.52	9.65 ± 0.47	8.74 ± 0.59
				L24	10.88 ± 0.49	9.50 ± 0.45	8.56 ± 0.56


*^*1*^Indicates the number of litters from each mating type (00/01/11); ^*2*^TNB, total number of born; L24, number of piglets alive at 24 h post-partum; and BA, number of piglets born live.*

### Candidate Gene Identification

A total of 95 known genes were identified around the detected haplotype blocks that were found associated with at least one of the fertility traits. Seventeen of them (Table 5) are involved in the functional pathways of fertility, disease or organ development, which might be related to early embryonic mortality or life survival. Their phenotypic effects, primarily drawn for studies of model organisms, are briefly described below. In previous studies, approaches that are similar to those used to obtain the results described above were also used to identify candidate loci that are essential for life in swine (Derks et al., 2017; Haggman and Uimari, 2017; Howard et al., 2017). However, in general, there was relatively little commonality of QTL across studies. To date, the populations explored have been independent and largely distinct from each other.

**Table 5:** Candidate genes occurring within 400 Kb flanking of a haplotype for which the minor allele homozygous genotype was not observed and that had an effect on at least one of the fertility traits.

Haplotype	SSC	Locus (Mb)	Gene name	Gene Start (bp)	Gene End (bp)	Distance (Kb)	Location
3	3	73.3–73.7	*CYP26B1*	73,915,121	73,931,838	215	Down-stream
8	5	20.1–20.5	*GTSF1*	20,494,143	20,504,958	0	Within
10–11	8	144.9–145.5	*SCD5*	144,868,128	145,004,045	0	Within
			*ENOPH1*	145,145,811	145,172,579	0	Within
13	9	19–19.4	*PCF11*	19,211,763	19,238,971	0	Within
14	9	126.9–127.3	*FASLG*	126,641,117	126,647,827	-259	Up-stream
			*TNFSF4*	127,044,587	127,046,516	0	Within
			*TNFSF18*	127,140,871	127,151,423	0	Within
15	10	61.8–62.2	*ITGB1*	61,450,695	61,502,941	-297	Up-stream
16	12	35.8–36.2	*TEX14*	35,849,615	36,000,403	0	Within
			*SEPT4*	36,030,882	36,043,170	0	Within
			*HSF5*	36,070,712	36,117,782	0	Within
			*RNF43*	36,120,600	36,181,669	0	Within
19	16	57.2–57.6	*FGF18*	56,921,614	56,956,559	-243	Up-stream
			*TLX3*	57,021,619	57,023,726	-176	Up-stream
			*GABRP*	57,477,086	57,495,797	0	Within
20–21	17	28.7–29.3	*PCSK2*	29,095,048	29,329,854	0	Within


x *CYP26B1* (Cytochrome P450 Family 26 Subfamily B Member 1) close to haplotype 3 on SSC3 is expressed in the testis of embryonic mice and plays a central role in germ cell development, but not in ovarian tissue of like-aged female embryos (Koubova et al., 2006; Kipp et al., 2011). In three *CYP26B1* mutant carrier x carrier families, *in utero* mortality of three individuals was observed in the first family, one patient in a second survived to 5 months of age, and in the third family an affected individual survived beyond infancy (Morton et al., 2016). *PCF11* (cleavage complex II protein) is within haplotype 13 on SSC9. The mutations in *PCF11* exhibit recessive lethality in Drosophila (Xie and Birchler, 2012). Haplotype 19 on SCC16 contains several important genes. *TLX3* (T Cell Leukemia Homeobox 3) is related to the pathways of sudden infant death syndrome (SIDS) susceptibility and transcriptional misregulation in cancer (Weese-Mayer et al., 2004). The subunit encoded by *GABRP* (Gamma-Aminobutyric Acid Type A Receptor Pi Subunit) is expressed in several non-neuronal tissues including the uterus and ovaries (Kim et al., 2015). The presence of this subunit alters the sensitivity of recombinant receptors to modulatory agents such as pregnanolone. In the uterus, the function of the receptor appears to be related to tissue contractility.

Several genes within or around the significant haplotypes are involved in spermiogenesis related pathways which may affect prenatal survival. Haplotype 16 around 35.8 – 36.3 Mb on SSC12 is relatively gene rich. *TEX14* (Testis Expressed 14, Intercellular Bridge Forming Factor) and *SEPT4* (Septin 4) showed significant effect on spermatogenesis and male fertility. Diseases associated with *TEX14* include Spermatogenic Failure 23. Formation of intercellular bridges in the testis of mice requires expression of *TEX14* (Greenbaum et al., 2006). These intercellular bridges are evolutionarily conserved structures that connect differentiating germ cells and are required for spermatogenesis and male fertility. *SEPT4* is probably required for the structural integrity and motility of the sperm tail during postmeiotic differentiation (Kuo et al., 2015). *SEPT4*-null mice have defects in caspase-mediated elimination of bulk cytoplasm during spermiogenesis and thus afflicted males are sterile but survive (Kissel et al., 2005; García-Fernández et al., 2010). *HSF5* (Heat Shock Transcription Factor 5) may act as a transcriptional factor. Testes of adult *HSF5*-/- male zebrafish have relatively abundant spermatocytes and very few spermatozoa resulting from miss-regulation of the cell cycle and meiosis (Orbán et al., 2018). *GTSF1* (Gametocyte Specific Factor 1) within haplotype 8 on SSC5 is required for spermatogenesis and is involved in the suppression of retrotransposon transcription in male germ cells.

A few genes in Haplotype 14 and Haplotype 17 are involved in tumor necrosis, e.g., *FASLG* (Fas Ligand), *TNFSF4* (TNF Superfamily Member 4) and *TNFSF18* (TNF Superfamily Member 18), or tumor suppression, e.g., *RNF43* (Ring Finger Protein 43) and *PCSK2* (Proprotein Convertase Subtilisin/Kexin Type 2). These genes are involved in important signaling pathways that are essential for immune system regulation, organ development, tumor development and progression. Mutations in these genes may affect essential functions resulting in more stillborn piglets. The protein encoded by *FGF18* (Fibroblast Growth Factor 18) is a member of the fibroblast growth factor (FGF) family which are involved in a variety of biological processes including embryonic development (Usui et al., 2004; Hung et al., 2016). A few genes associated with disease were also found in this study. Both *SCD5* (Stearoyl-CoA Desaturase 5) and *ENOPH1* (Enolase-Phosphatase 1) within Haplotypes 10-11 on SSC8 are related to the disease of Chromosome 4Q21 Deletion Syndrome. Mouse pups that do not express *SCD5* are stillborn, with reduced growth rate *in utero* (Krakowiak et al., 2003). Diseases associated with *ITGB1* (Integrin Subunit Beta 1) include Gallbladder Cancer and Breast Cancer. Integrin family members are membrane receptors involved in a variety of processes including embryogenesis, hemostasis, tissue repair, immune response and metastatic diffusion of tumor cells.

For haplotype 4 on SSC3, no known gene was detected around this block. However, a few interesting genes (not shown in Table 5) around 1–3 Mb of this block were involved in some important pathways of reproduction or life. *FSHR* is the receptor for follicle stimulating hormone and functions in gonad development (Costagliola et al., 2002). Mutations in this gene cause ovarian dysgenesis type 1 and ovarian hyperstimulation syndrome (Uchida et al., 2013). *EPCAM* (Epithelial Cell Adhesion Molecule) plays a role in embryonic stem cell proliferation and differentiation. Mutations in *CALM2* (Calmodulin 2) or another member of the calmodulin gene family seem to result in patients with severe forms of long-QT syndrome (LQTS) who displayed life-threatening ventricular arrhythmias, a rare disorder thought to be the cause of a significant fraction of sudden cardiac deaths in young individuals, and together with delayed neurodevelopment and epilepsy (Nyegaard et al., 2012; Crotti et al., 2013; Makita et al., 2014).

## Conclusion

These findings facilitate potentially increasing Duroc litter size through a marker-assisted breeding and mate selection designed to avoid producing homozygous zygotes. If these technologies can be successfully implemented, breeders should benefit from increased selection intensity as well as having an increased number of pigs to market from each litter. In addition, the results serve to validate previous findings as to loci which affect prenatal mortality and consequently litter size. The identified candidate genes may also lead to new insights for the biology of pig fertility.

## Ethics Statement

Data and samples for genotyping were collected at Genesus Inc. Research and Development Facilities (Genesus Inc., Oakville, MB, Canada) as part of routine commercial operation prior to the research. The proposed work was reviewed by the University of Alberta Animal Care and Use Committee. No other specific permissions were required for the work, since the animals were cared for according to the Canadian Quality Assurance Program, which includes attention to animal health and well-being and is in line with the Canadian Council on Animal Care guidelines.

## Author Contributions

GP, MM, and MD conceived and designed the experiments. CZ performed the statistical analysis. GP and MM provided valuable advice to refine the statistical analyses. RK contributed materials and application advice. CZ, GP, and MM were major contributors in writing the manuscript. All authors read and approved the final manuscript.

## Conflict of Interest Statement

RK is employed by Genesus Inc. who provided data and genotypes as an in kind contribution to the research. RK was involved in the sample and data collection and contributed to the paper by advising on the potential application of the results. The potential conflict of interest did not interfere with the outcome of this paper. The remaining authors declare that the research was conducted in the absence of any commercial or financial relationships that could be construed as a potential conflict of interest.

## Footnotes

1 http://bioinformatics.tecnoparco.org/SNPchimp/index.php/data-source

2 http://www.ensembl.org/biomart/martview

3 http://www.genecards.org/

## Supplementary Material

TABLE S1 Location and minor allele frequency for haplotypes which were expected to have two homozygous genotypes, but with only one found in the data and which did not have a significant effect on any of the fertility phenotypes. ^1^Number of SNPs in the haplotype blocks; ^2^Haplotype index; ^3^number of individuals that were expected to be homozygous for the minor allele; ^4^Bonferroni corrected *P*-value.
