# Supplementary material for: Putative Loci Causing Early Embryonic Mortality in Duroc Swine
Source: Front Genet. 2018 Dec 17;9:655. doi: 10.3389/fgene.2018.00655 (PMC6304751; doi:10.3389/fgene.2018.00655)
Supplement: TABLE S1 — Location and minor allele frequency for haplotypes which were expected to have two homozygous genotypes, but with only one found in the data and which did not have a significant effect on any of the fertility phenotypes. 1Number of SNPs in the haplotype blocks; 2Haplotype index; 3number of individuals that were expected to be homozygous for the minor allele; 4Bonferroni corrected P-value. [file Table_1.docx]

| Table S1. Location and minor allele frequency for haplotypes which were expected to have two homozygous genotypes, but with only one found in the data and which did not have a significant effect on any of the fertility phenotypes. | | | | | | |
| --- | --- | --- | --- | --- | --- | --- |
| **SSC** | **Locus (Mb)** | **# SNPs^1^** | **Haplotype^2^** | **Frequency** | **E(HOM)^3^** | **P^4^** |
| 1 | 54.6-55 | 15 | 22 | 0.15 | 36 | 5.7E-11 |
| 1 | 54.6-55 | 15 | 23 | 0.12 | 23 | 2.1E-05 |
| 1 | 54.7-55.1 | 18 | 24 | 0.15 | 33 | 1.5E-09 |
| 1 | 54.7-55.1 | 18 | 25 | 0.12 | 24 | 8.8E-06 |
| 1 | 92.4-92.8 | 12 | 26 | 0.16 | 39 | 2.5E-12 |
| 1 | 97.2-97.6 | 25 | 27 | 0.14 | 32 | 3.6E-09 |
| 1 | 97.3-97.7 | 20 | 28 | 0.14 | 32 | 3.6E-09 |
| 1 | 135.2-135.6 | 33 | 29 | 0.13 | 25 | 2.2E-06 |
| 1 | 135.2-135.6 | 33 | 30 | 0.11 | 17 | 6.4E-03 |
| 1 | 135.3-135.7 | 38 | 31 | 0.13 | 27 | 3.5E-07 |
| 1 | 135.3-135.7 | 38 | 32 | 0.11 | 19 | 1.6E-03 |
| 1 | 137.6-138 | 56 | 33 | 0.12 | 24 | 1.0E-05 |
| 1 | 137.6-138 | 56 | 34 | 0.11 | 19 | 1.7E-03 |
| 1 | 137.7-138.1 | 66 | 35 | 0.12 | 23 | 1.3E-05 |
| 1 | 137.7-138.1 | 66 | 36 | 0.12 | 20 | 2.5E-04 |
| 2 | 4.5-4.9 | 27 | 37 | 0.11 | 20 | 5.7E-04 |
| 2 | 4.5-4.9 | 27 | 38 | 0.11 | 19 | 7.1E-04 |
| 2 | 4.6-5 | 24 | 39 | 0.11 | 19 | 7.1E-04 |
| 2 | 4.6-5 | 24 | 40 | 0.11 | 19 | 7.1E-04 |
| 2 | 47.2-47.6 | 79 | 41 | 0.11 | 17 | 6.4E-03 |
| 3 | 73.2-73.6 | 29 | 42 | 0.11 | 20 | 6.4E-04 |
| 3 | 73.4-73.8 | 35 | 43 | 0.11 | 18 | 3.0E-03 |
| 3 | 84.9-85.3 | 20 | 44 | 0.11 | 19 | 1.0E-03 |
| 3 | 95.9-96.3 | 59 | 45 | 0.11 | 20 | 5.1E-04 |
| 3 | 96-96.4 | 52 | 46 | 0.12 | 22 | 5.5E-05 |
| 4 | 20.6-21 | 9 | 47 | 0.11 | 17 | 6.4E-03 |
| 4 | 27.1-27.5 | 31 | 48 | 0.13 | 26 | 6.8E-07 |
| 4 | 27.1-27.5 | 31 | 49 | 0.13 | 26 | 8.8E-07 |
| 4 | 27.2-27.6 | 39 | 50 | 0.12 | 21 | 1.4E-04 |
| 4 | 27.2-27.6 | 39 | 51 | 0.11 | 18 | 1.9E-03 |
| 4 | 46.5-46.9 | 16 | 52 | 0.18 | 48 | 2.4E-16 |
| 4 | 46.7-47.1 | 38 | 53 | 0.11 | 18 | 2.7E-03 |
| 4 | 46.9-47.3 | 52 | 54 | 0.11 | 18 | 3.3E-03 |
| 4 | 58.8-59.2 | 56 | 55 | 0.11 | 19 | 1.7E-03 |
| 4 | 74.7-75.1 | 9 | 56 | 0.12 | 24 | 8.8E-06 |
| 4 | 99.6-100 | 51 | 57 | 0.12 | 20 | 2.5E-04 |
| 4 | 99.6-100 | 51 | 58 | 0.11 | 18 | 4.1E-03 |
| 5 | 3.6-4 | 26 | 59 | 0.11 | 20 | 4.5E-04 |
| 5 | 13.6-14 | 25 | 60 | 0.11 | 19 | 8.9E-04 |
| 5 | 20-20.4 | 17 | 61 | 0.13 | 27 | 3.1E-07 |
| 6 | 6.2-6.6 | 20 | 62 | 0.15 | 33 | 8.5E-10 |
| 6 | 6.3-6.7 | 18 | 63 | 0.18 | 47 | 6.8E-16 |
| 6 | 6.4-6.8 | 37 | 64 | 0.17 | 46 | 2.0E-15 |
| 6 | 6.5-6.9 | 37 | 65 | 0.16 | 39 | 1.8E-12 |
| 6 | 111.1-111.5 | 34 | 66 | 0.11 | 18 | 3.0E-03 |
| 6 | 111.2-111.6 | 34 | 67 | 0.11 | 19 | 7.1E-04 |
| 6 | 111.3-111.7 | 46 | 68 | 0.12 | 21 | 1.6E-04 |
| 7 | 60.8-61.2 | 13 | 69 | 0.24 | 85 | 1.6E-32 |
| 7 | 60.8-61.2 | 13 | 70 | 0.20 | 59 | 6.9E-21 |
| 7 | 60.9-61.3 | 11 | 71 | 0.24 | 91 | 8.2E-35 |
| 7 | 60.9-61.3 | 11 | 72 | 0.21 | 67 | 1.1E-24 |
| 7 | 83.3-83.7 | 38 | 73 | 0.12 | 21 | 1.8E-04 |
| 7 | 83.4-83.8 | 36 | 74 | 0.12 | 21 | 1.8E-04 |
| 7 | 83.5-83.9 | 14 | 75 | 0.12 | 21 | 1.1E-04 |
| 7 | 84.1-84.5 | 23 | 76 | 0.13 | 26 | 1.5E-06 |
| 7 | 84.4-84.8 | 21 | 77 | 0.10 | 17 | 9.7E-03 |
| 7 | 85.7-86.1 | 5 | 78 | 0.11 | 17 | 8.7E-03 |
| 7 | 109.6-110 | 24 | 79 | 0.12 | 22 | 4.3E-05 |
| 8 | 28.3-28.7 | 14 | 80 | 0.11 | 18 | 4.1E-03 |
| 8 | 49.5-49.9 | 7 | 81 | 0.11 | 18 | 3.0E-03 |
| 9 | 39.1-39.5 | 13 | 82 | 0.11 | 18 | 2.4E-03 |
| 9 | 57.9-58.3 | 21 | 83 | 0.15 | 33 | 7.3E-10 |
| 9 | 124.8-125.2 | 13 | 84 | 0.12 | 24 | 1.0E-05 |
| 9 | 126.7-127.1 | 21 | 85 | 0.16 | 37 | 1.7E-11 |
| 10 | 29-29.4 | 36 | 86 | 0.12 | 23 | 3.0E-05 |
| 10 | 29.1-29.5 | 38 | 87 | 0.11 | 19 | 8.9E-04 |
| 10 | 51.3-51.7 | 46 | 88 | 0.13 | 27 | 3.1E-07 |
| 10 | 51.3-51.7 | 46 | 89 | 0.11 | 17 | 7.1E-03 |
| 10 | 51.4-51.8 | 71 | 90 | 0.14 | 30 | 2.6E-08 |
| 10 | 51.4-51.8 | 71 | 91 | 0.11 | 17 | 6.4E-03 |
| 10 | 51.5-51.9 | 78 | 92 | 0.14 | 28 | 9.1E-08 |
| 10 | 51.6-52 | 83 | 93 | 0.12 | 21 | 1.0E-04 |
| 11 | 17.6-18 | 7 | 94 | 0.19 | 56 | 8.5E-20 |
| 11 | 17.7-18.1 | 10 | 95 | 0.12 | 22 | 7.0E-05 |
| 11 | 62.5-62.9 | 12 | 96 | 0.12 | 21 | 2.3E-04 |
| 12 | 35.9-36.3 | 76 | 97 | 0.11 | 17 | 7.1E-03 |
| 12 | 37-37.4 | 32 | 98 | 0.15 | 35 | 1.0E-10 |
| 14 | 32.8-33.2 | 45 | 99 | 0.13 | 27 | 4.6E-07 |
| 14 | 59.1-59.5 | 73 | 100 | 0.12 | 24 | 1.0E-05 |
| 14 | 59.2-59.6 | 76 | 101 | 0.12 | 21 | 1.3E-04 |
| 14 | 59.3-59.7 | 85 | 102 | 0.11 | 18 | 2.7E-03 |
| 14 | 87.1-87.5 | 25 | 103 | 0.11 | 18 | 2.4E-03 |
| 14 | 148.4-148.8 | 13 | 104 | 0.11 | 17 | 7.1E-03 |
| 15 | 14.5-14.9 | 6 | 105 | 0.11 | 19 | 8.9E-04 |
| 15 | 16.7-17.1 | 50 | 106 | 0.11 | 19 | 1.4E-03 |
| 15 | 73-73.4 | 37 | 107 | 0.12 | 23 | 1.8E-05 |
| 15 | 73.1-73.5 | 28 | 108 | 0.16 | 39 | 2.9E-12 |
| 15 | 73.2-73.6 | 30 | 109 | 0.16 | 41 | 2.2E-13 |
| 15 | 73.3-73.7 | 36 | 110 | 0.16 | 41 | 4.3E-13 |
| 15 | 75-75.4 | 19 | 111 | 0.13 | 27 | 5.2E-07 |
| 15 | 76-76.4 | 32 | 112 | 0.12 | 23 | 3.0E-05 |
| 15 | 76-76.4 | 32 | 113 | 0.12 | 21 | 2.0E-04 |
| 15 | 76.1-76.5 | 38 | 114 | 0.12 | 22 | 3.4E-05 |
| 15 | 95.7-96.1 | 25 | 115 | 0.12 | 23 | 3.0E-05 |
| 15 | 95.8-96.2 | 24 | 116 | 0.12 | 22 | 7.9E-05 |
| 15 | 95.9-96.3 | 14 | 117 | 0.12 | 22 | 5.5E-05 |
| 15 | 96-96.4 | 21 | 118 | 0.14 | 28 | 1.2E-07 |
| 15 | 96-96.4 | 21 | 119 | 0.13 | 24 | 5.3E-06 |
| 15 | 110.1-110.5 | 11 | 120 | 0.13 | 26 | 1.0E-06 |
| 15 | 110.1-110.5 | 11 | 121 | 0.11 | 19 | 8.0E-04 |
| 15 | 110.2-110.6 | 16 | 122 | 0.12 | 22 | 7.0E-05 |
| 15 | 118.4-118.8 | 25 | 123 | 0.11 | 19 | 1.0E-03 |
| 16 | 7.5-7.9 | 8 | 124 | 0.11 | 17 | 6.4E-03 |
| 16 | 9.9-10.3 | 20 | 125 | 0.20 | 62 | 2.4E-22 |
| 16 | 10-10.4 | 27 | 126 | 0.11 | 20 | 5.7E-04 |
| 16 | 41.6-42 | 73 | 127 | 0.13 | 24 | 7.8E-06 |
| 16 | 41.7-42.1 | 57 | 128 | 0.13 | 25 | 2.5E-06 |
| 16 | 41.8-42.2 | 63 | 129 | 0.13 | 24 | 6.8E-06 |
| 16 | 41.8-42.2 | 63 | 130 | 0.11 | 17 | 5.1E-03 |
| 16 | 41.9-42.3 | 69 | 131 | 0.13 | 25 | 1.7E-06 |
| 16 | 41.9-42.3 | 69 | 132 | 0.11 | 18 | 4.1E-03 |
| 16 | 49.7-50.1 | 24 | 133 | 0.19 | 54 | 5.7E-19 |
| 16 | 49.7-50.1 | 24 | 134 | 0.11 | 18 | 3.0E-03 |
| 16 | 49.8-50.2 | 10 | 135 | 0.19 | 56 | 5.8E-20 |
| 16 | 49.8-50.2 | 10 | 136 | 0.12 | 22 | 3.4E-05 |
| 16 | 54.5-54.9 | 5 | 137 | 0.14 | 29 | 5.3E-08 |
| 16 | 56-56.4 | 28 | 138 | 0.11 | 18 | 2.4E-03 |
| 16 | 56.1-56.5 | 29 | 139 | 0.11 | 20 | 5.1E-04 |
| 16 | 56.9-57.3 | 35 | 140 | 0.13 | 24 | 4.7E-06 |
| 16 | 57-57.4 | 24 | 141 | 0.13 | 25 | 2.5E-06 |
| 16 | 57.1-57.5 | 7 | 142 | 0.17 | 45 | 5.5E-15 |
| 17 | 38.3-38.7 | 37 | 143 | 0.12 | 22 | 3.4E-05 |
| 17 | 52.2-52.6 | 12 | 144 | 0.13 | 28 | 2.1E-07 |
| 17 | 55.1-55.5 | 46 | 145 | 0.13 | 28 | 2.1E-07 |
| 17 | 55.2-55.6 | 37 | 146 | 0.14 | 29 | 7.9E-08 |
| 17 | 56.5-56.9 | 53 | 147 | 0.14 | 30 | 1.7E-08 |
| 17 | 57.7-58.1 | 32 | 148 | 0.15 | 34 | 4.7E-10 |
| 17 | 57.8-58.2 | 26 | 149 | 0.25 | 97 | 1.3E-37 |
| 17 | 57.9-58.3 | 21 | 150 | 0.25 | 97 | 1.0E-37 |
| 17 | 58-58.4 | 28 | 151 | 0.26 | 102 | 1.0E-39 |
| 17 | 63-63.4 | 81 | 152 | 0.12 | 23 | 1.4E-05 |
